# Supplementary material for: Livestock, pathogens, vectors, and their environment: A causal inference-based approach to estimating the pathway-specific effect of livestock on human African trypanosomiasis risk
Source: PLOS Glob Public Health. 2023 Nov 15;3(11):e0002543. doi: 10.1371/journal.pgph.0002543 (PMC10651035; doi:10.1371/journal.pgph.0002543)
Supplement: S3 Appendix — (PDF) [file pgph.0002543.s003.pdf]

S4 Appendix: Descriptive statistics plots

Malawi

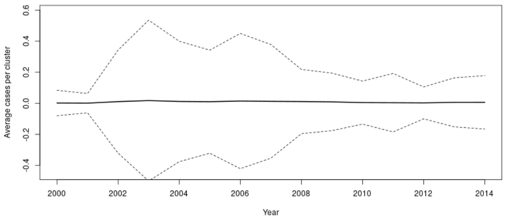

(a)

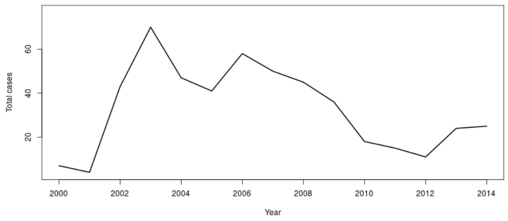

(b)

Figure 4.1: Mean (a) and sum (b) of cases over time in study clusters, Malawi

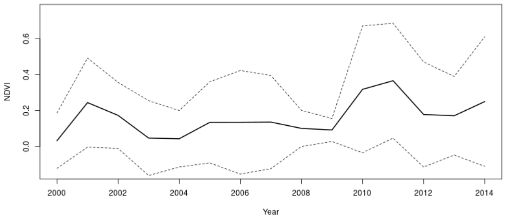

(a) NDVI

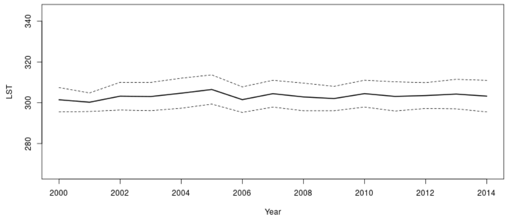

(b) LST

Figure 4.2: NDVI (a) and LST (b) over time in study clusters, Malawi

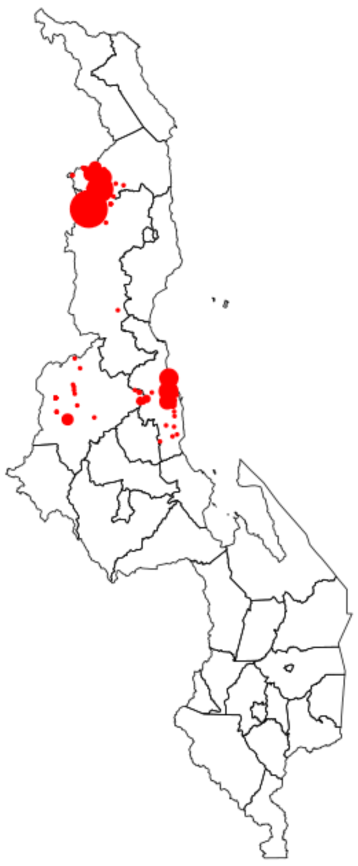

Figure 4.3: HAT cases 2000-2014, Malawi. All base maps were obtained from GADM https://geodata.ucdavis.edu/gadm/gadm4.1/shp/gadm41MW Ishp.zip)

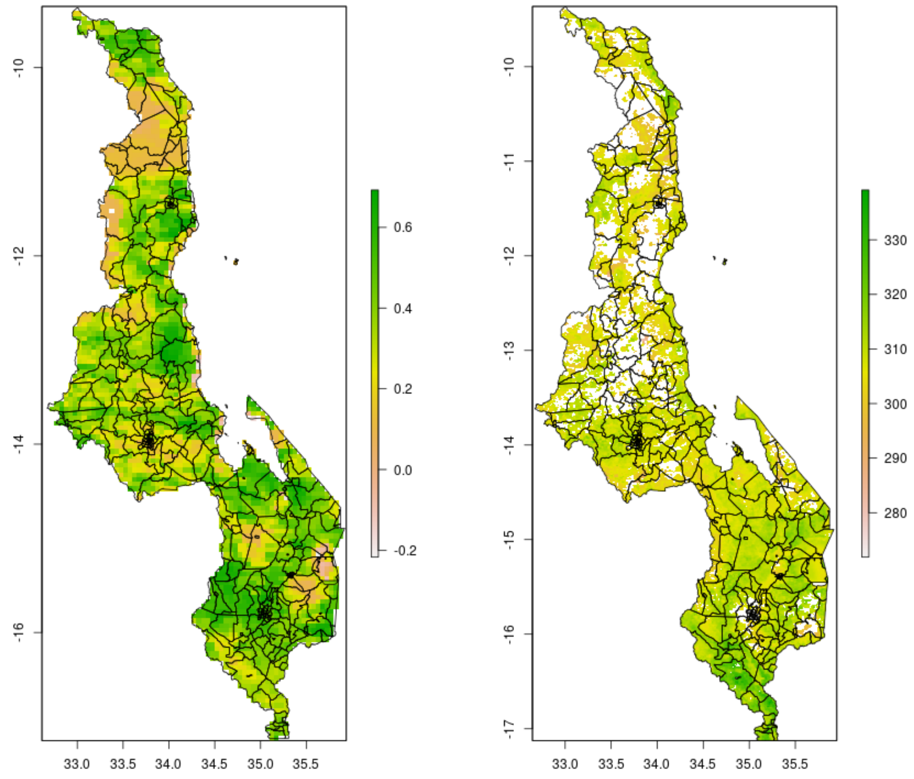

(a) (b)

Figure 4.4: 2010 NDVI (a) and LST (b), Malawi. All base maps were obtained from GADM https://geodata.ucdavis.edu/gadm/gadm4.1/shp/gadm41MW Ishp.zip)

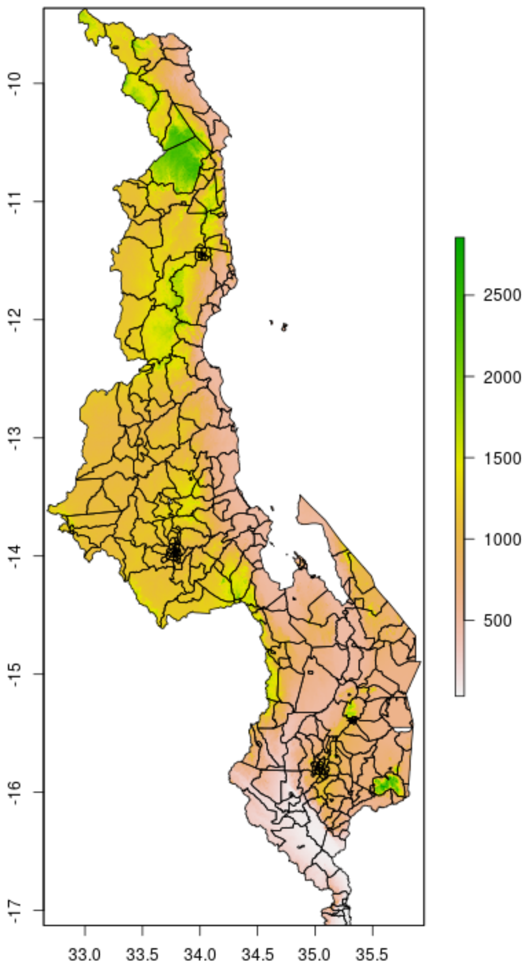

Figure 4.5: Elevation, Malawi. All base maps were obtained from GADM https://geodata.ucdavis.edu/gadm/gadm4.1/shp/gadm41MW Ishp.zip)

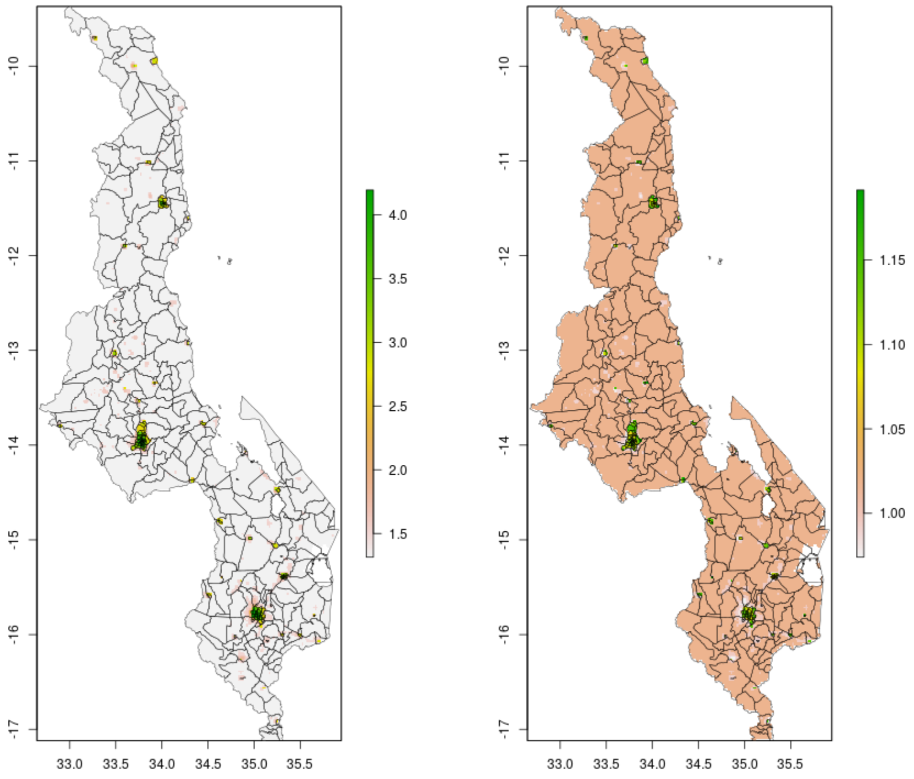

(a) (b)

Figure 4.6: 2010 wealth scores, mean (a) and posterior 95% credible interval (b), Malawi. All base maps were obtained from GADM https://geodata.ucdavis.edu/gadm/gadm4.1/shp/gadm41MW Ishp.zip)

Uganda

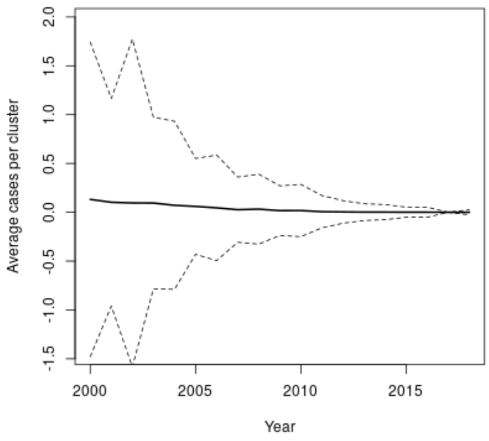

(a) gHAT

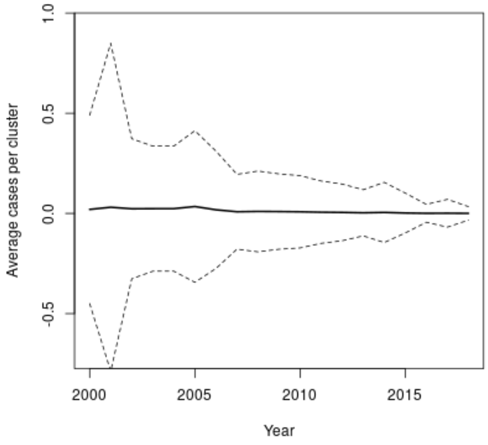

(b) rHAT

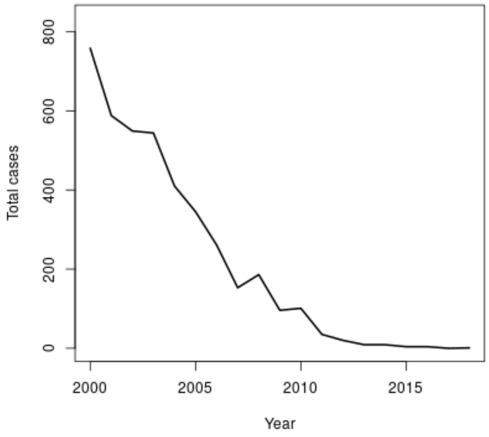

(c) gHAT

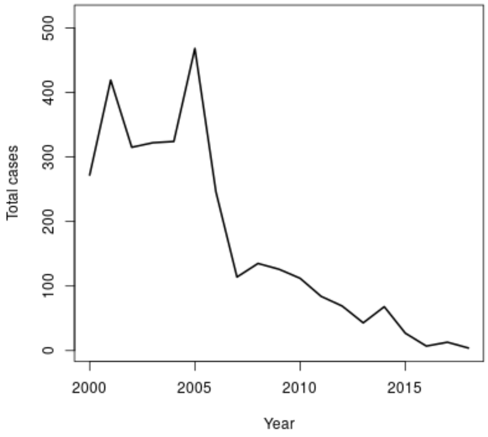

(d) rHAT

Figure 4.7: Mean (a-b) and (c-d) of cases over time in study clusters, Uganda

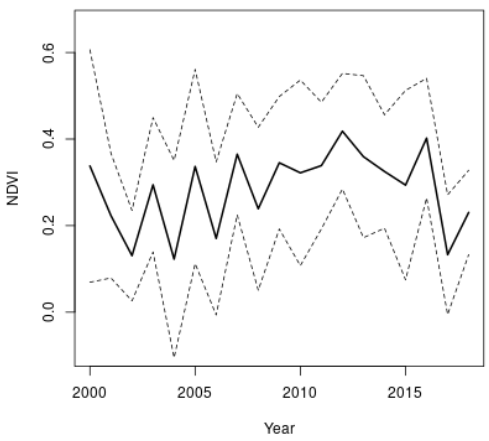

(a) gHAT

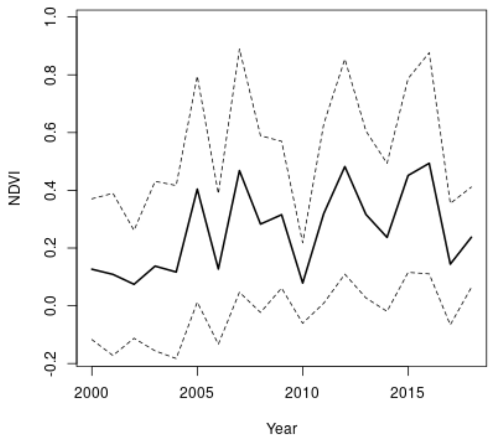

(b) rHAT

Figure 4.8: NDVI over time in study clusters, Uganda

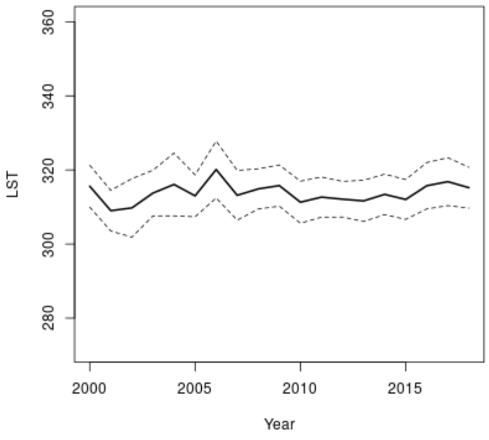

(a) gHAT

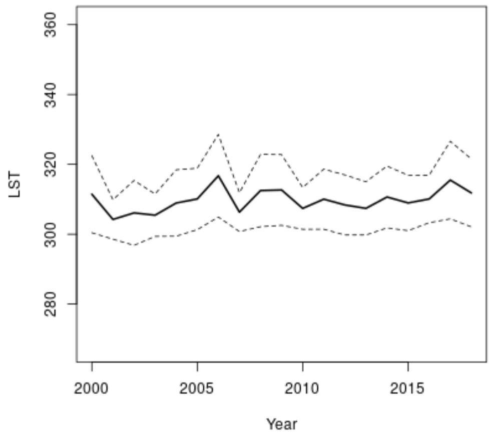

(b) rHAT

Figure 4.9: LST over time in study clusters, Uganda

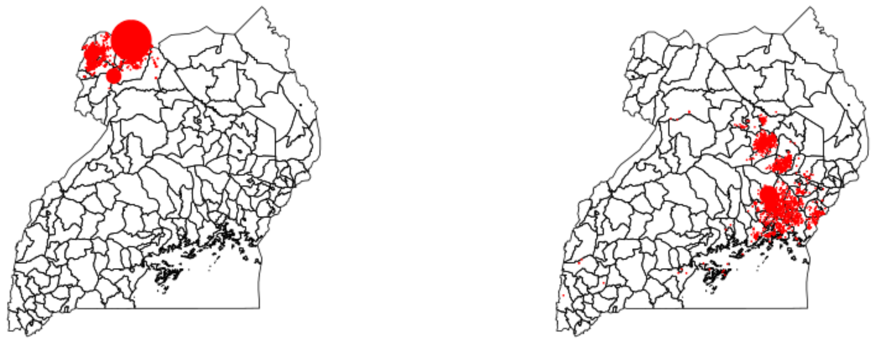

(a) gHAT (b) rHAT

Figure 4.10: HAT cases 2000-2018, Uganda. All base maps were obtained from GADM (https : //geodata.ucdavis.edu/gadm/gadm4.1/shp/gadm41UGAshp.zip)

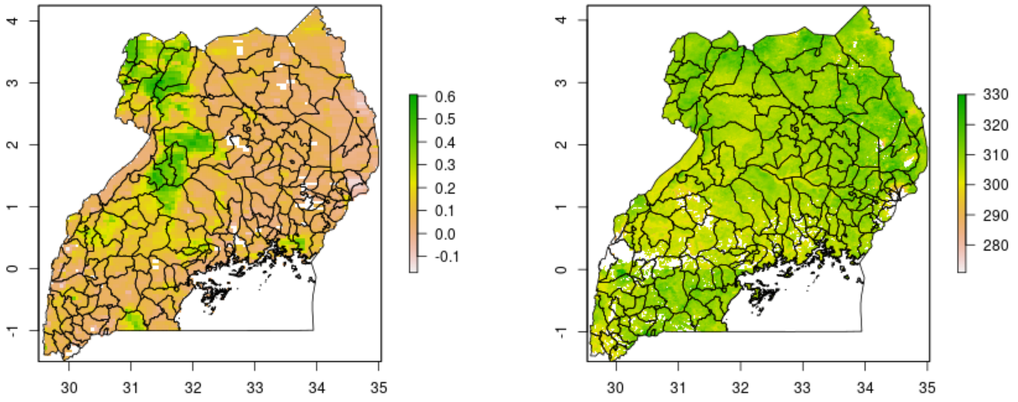

(a) (b)

Figure 4.11: 2010 NDVI (a) and LST (b), Uganda. All base maps were obtained from GADM (https : //geodata.ucdavis.edu/gadm/gadm4.1/shp/gadm41UGAshp.zip)

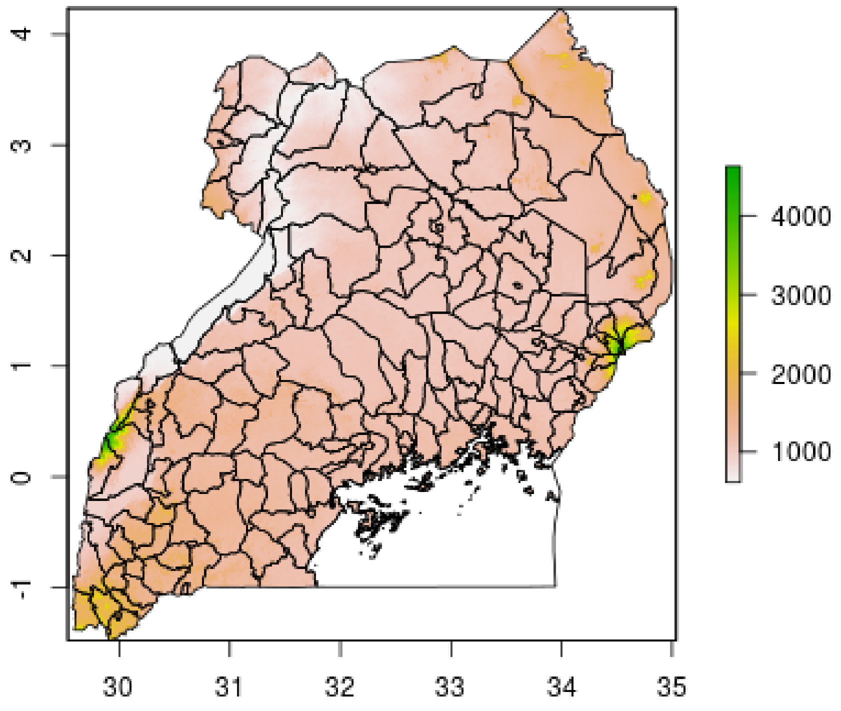

Figure 4.12: Elevation, Uganda. All base maps were obtained from GADM (https : //geo-data.ucdavis.edu/gadm/gadm4.1/shp/gadm41UGAshp.zip)

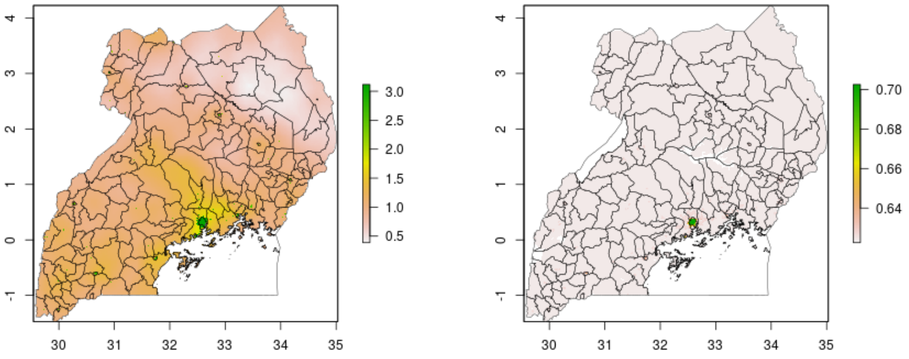

(a) (b)

Figure 4.13: 2010 wealth scores, mean (a) and posterior 95% credible interval (b), Uganda. All base maps were obtained from GADM (https : //geodata.ucdavis.edu/gadm/gadm4.1/shp/gadm41UGAshp.zip)

DRC

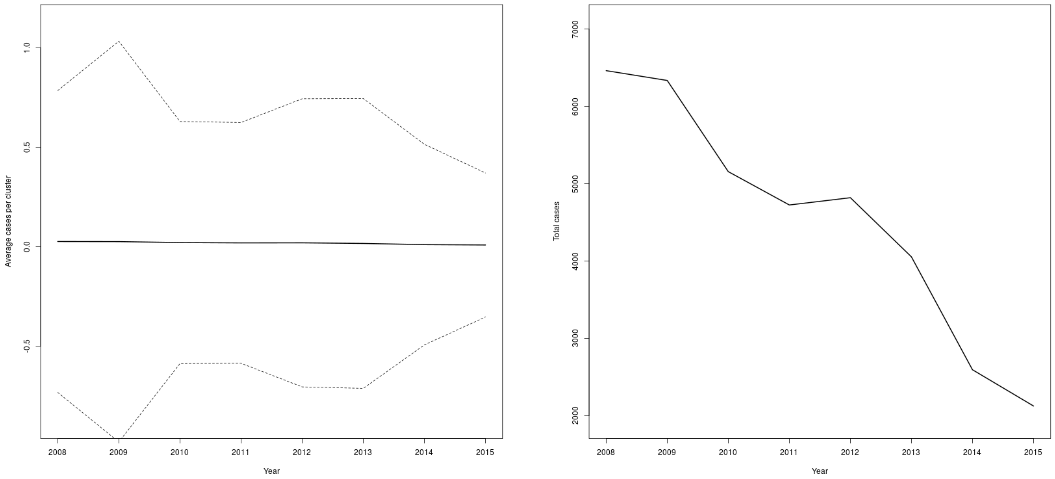

(a) Mean (b) Sum

Figure 4.14: Mean (a) and sum (b) of cases over time in study clusters, DRC

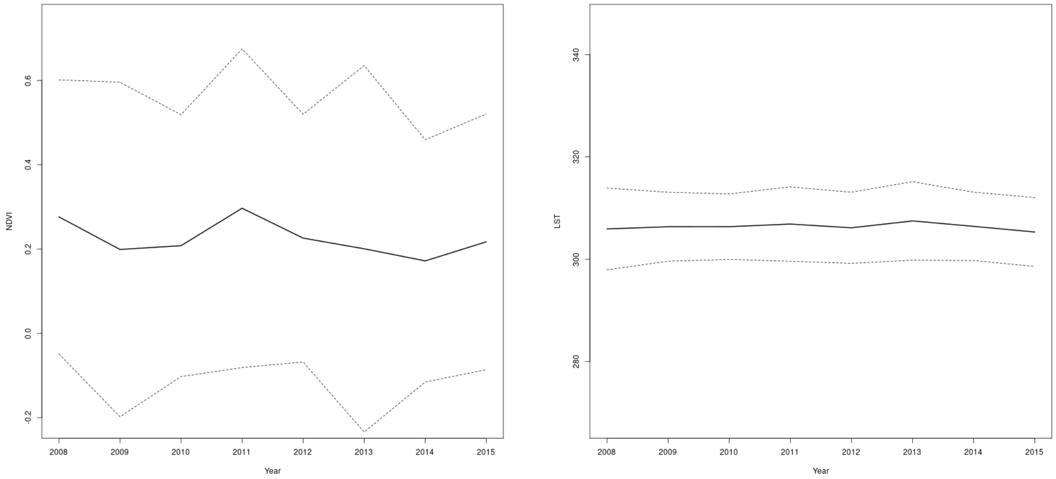

(a) NDVI

(b) LST

Figure 4.15: NDVI (a) and LST (b) over time in study clusters, DRC

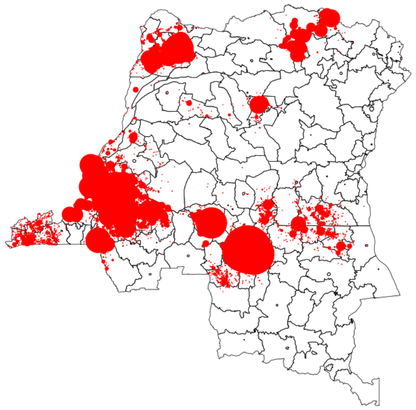

Figure 4.16: HAT cases 2008-2015, DRC. All base maps were obtained from GADM (https : //geodata.ucdavis.edu/gadm/gadm4.1/shp/gadm41C ODshp.zip)

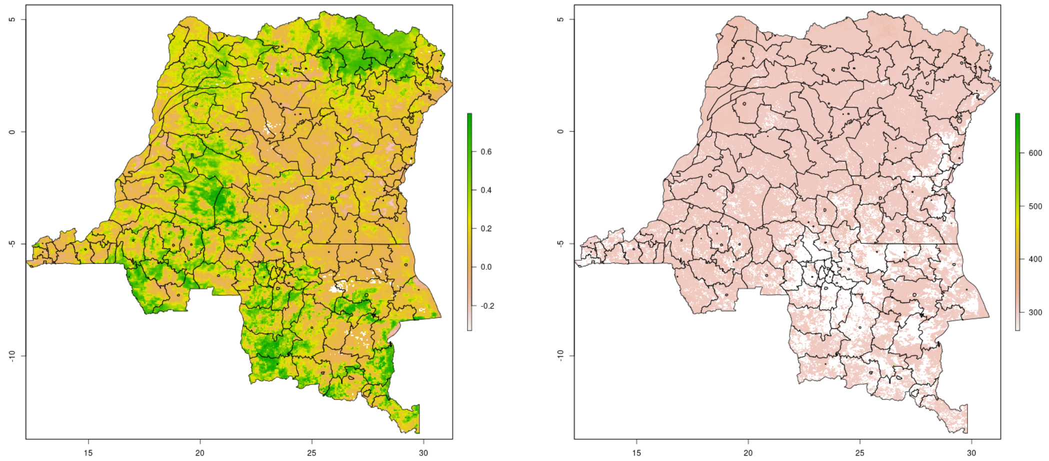

(a) (b)

Figure 4.17: 2010 NDVI (a) and LST (b), DRC. All base maps were obtained from GADM (https : //geodata.ucdavis.edu/gadm/gadm4.1/shp/gadm41C ODshp.zip)

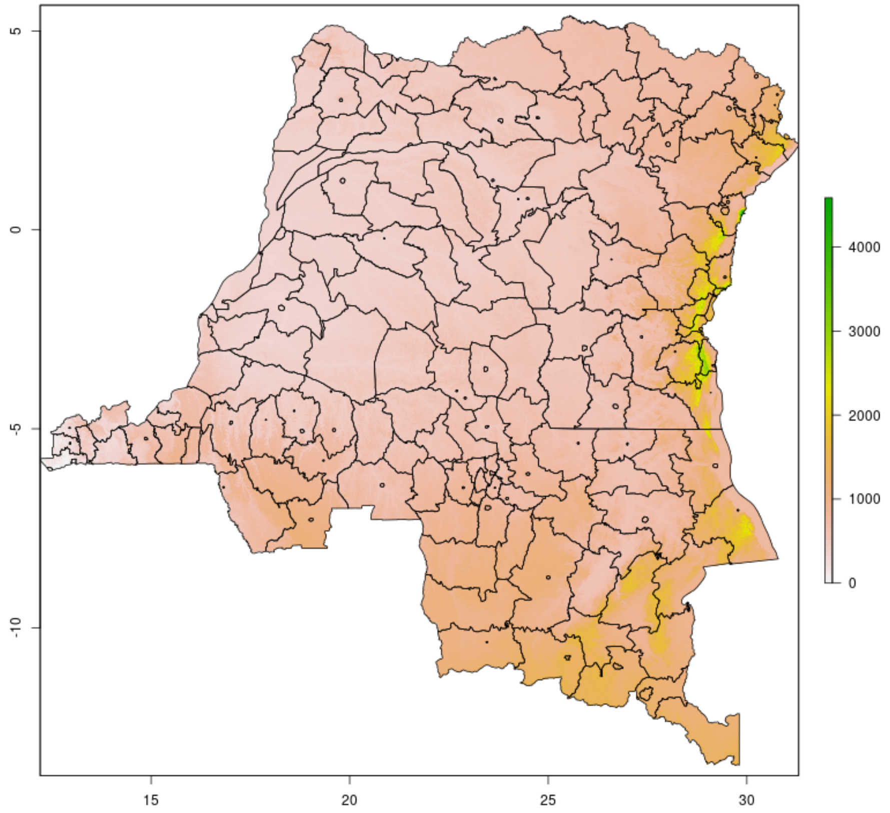

Figure 4.18: Elevation, DRC. All base maps were obtained from GADM (https : //geodata.ucdavis.edu/gadm/gadm4.1/shp/gadm41C ODshp.zip)

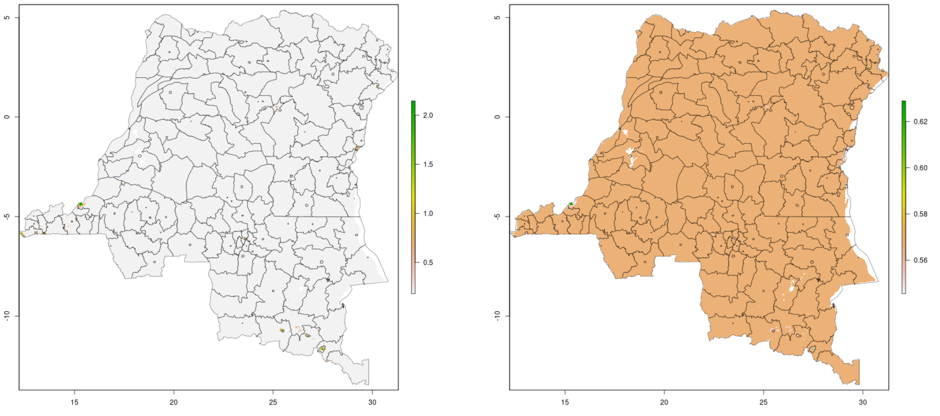

(a) (b)

Figure 4.19: 2010 wealth scores, mean (a) and posterior 95% credible interval (b), DRC. All base maps were obtained from GADM (https : //geodata.ucdavis.edu/gadm/gadm4.1/shp/gadm41C ODshp.zip)

South Sudan

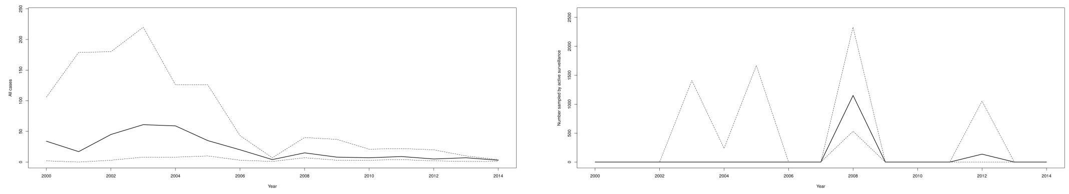

(a) (b)

Figure 4.20: Mean number of cases (a) and people sampled by active surveillance (b) over time in study counties, South Sudan

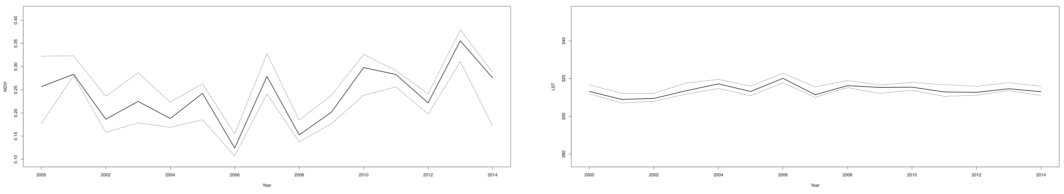

(a) NDVI (b) LST

Figure 4.21: NDVI (a) and LST (b) over time in study counties, South Sudan

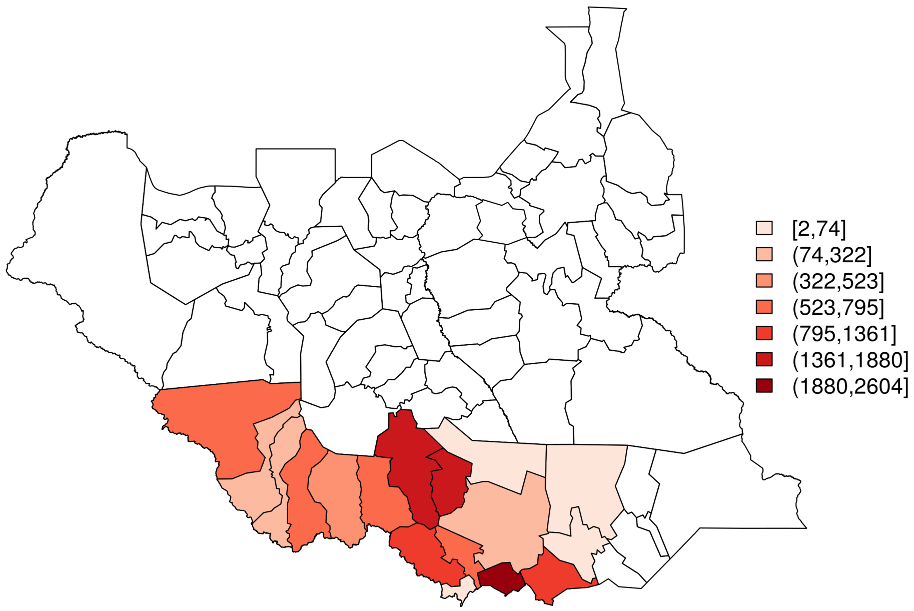

Figure 4.22: HAT cases 2000-2014, South Sudan. All base maps were obtained from GADM (SouthSudan : https : //geodata.ucdavis.edu/gadm/gadm4.1/shp/gadm41SSDshp.zip)

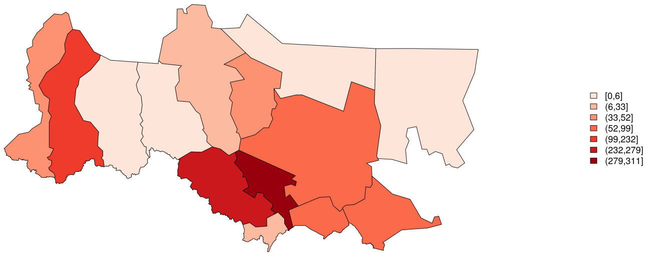

(a) Cases detected

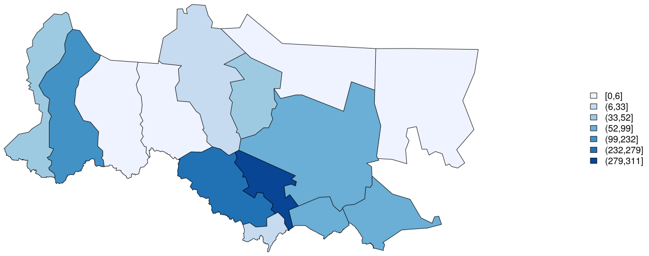

(b) Number sampled

Figure 4.23: Total cases detected (a) and number sampled (b) by active surveillance in South Sudan, 2008 (restricted to active surveillance area). All base maps were obtained from GADM (SouthSudan : https : //geodata.ucdavis.edu/gadm/gadm4.1/shp/gadm41SSDshp.zip)

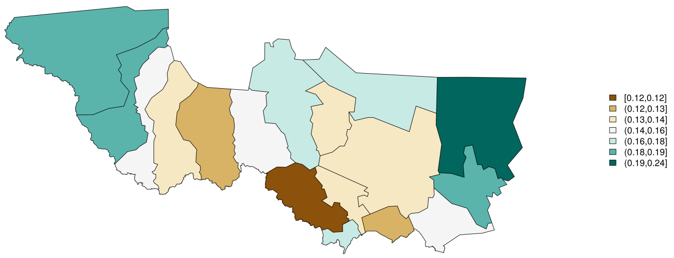

(a) NDVI

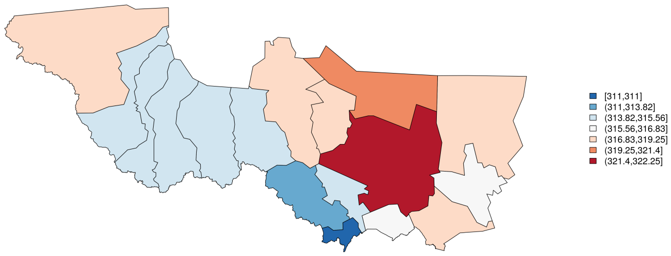

(b) LST

Figure 4.24: 2008 NDVI (a) and LST (b), South Sudan (study area). All base maps were obtained from GADM (SouthSudan : https : //geo-data.ucdavis.edu/gadm/gadm4.1/shp/gadm41SSDshp.zip)

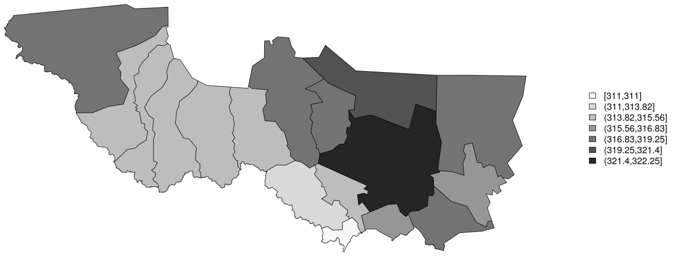

Figure 4.25: Elevation, South Sudan (study counties). All base maps were obtained from GADM (SouthSudan : https : //geo-data.ucdavis.edu/gadm/gadm4.1/shp/gadm41SSDshp.zip)

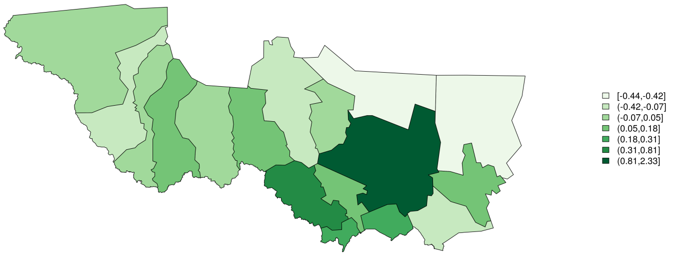

(a) Median

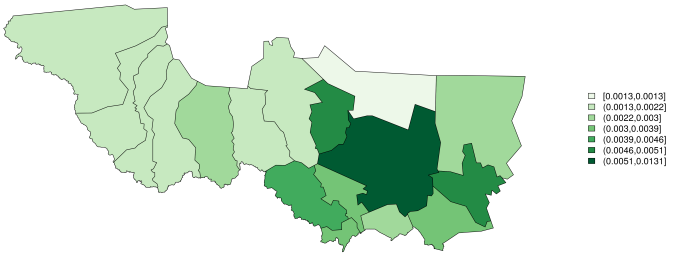

(b) Standard error

Figure 4.26: 2008 wealth, median (a) and standard error (b), South Sudan (study counties). All base maps were obtained from GADM (SouthSudan : https : //geo-data.ucdavis.edu/gadm/gadm4.1/shp/gadm41SSDshp.zip)
