# Supplementary material for: Livestock, pathogens, vectors, and their environment: A causal inference-based approach to estimating the pathway-specific effect of livestock on human African trypanosomiasis risk
Source: PLOS Glob Public Health. 2023 Nov 15;3(11):e0002543. doi: 10.1371/journal.pgph.0002543 (PMC10651035; doi:10.1371/journal.pgph.0002543)
Supplement: S4 Appendix — (PDF) [file pgph.0002543.s004.pdf]

S3 Appendix: Implementation of the measurement error model in South Sudan

The measurement error (MEC) model is defined as:

$$w_c = x_c + u_c, c = 1, ..., n$$

where c indexes county, u_c are the measurement error terms distributed as $u_c \sim N(0, \sigma_u^2)$, and x_c and u_c are independent. Since the u terms have mean 0, $E(W|X = x) = x$, that is, W is unbiased for a given unobserved x. Failing to account for measurement error will result in biased effect estimates and inappropriate standard errors [1].

Following the heteroscedastic errors-in-variables approach detailed in Wang et al. (2018), we defined our hierarchical model as follows:

$$\lambda = exp\Big(\theta_0 + \theta_1\mu_c + \theta_2 m_c + \theta_3\eta_c + \boldsymbol{\alpha}\mathbf{z}(\mathbf{k}_c) + S_c + \epsilon_c + log(P_c)\Big)$$

$$\mu_c = x_{lc} + u_{lc}, \eta_c = x_{wc} + u_{wc}$$

$$x_{lc} = \lambda_l + \zeta_{lc}, x_{wc} = \lambda_w + \zeta_{wc}$$

$$u_{lc} \sim N(0, d_c\sigma_{ul}^2), u_{wc} \sim N(0, d_c\sigma_{uw}^2)$$

$$\zeta_{lc} \sim N(0, \sigma_{xl}^2), \zeta_{wc} \sim N(0, \sigma_{xw}^2)$$

$$S_c|S_k, k \in ne(c) \sim N\left(\overline{S}_k, \frac{\sigma_s^2}{n_c}\right)$$

$$\epsilon_c|\sigma_\epsilon^2 \sim_{iid} N(0, \sigma_\epsilon^2)$$

where

- μ_c is estimated livestock density in county c in 2008
- m_c is the mediator value in county c in 2010, M ∈{NDVI, LST}
- η_c is estimated wealth score in county c in 2008
- $\boldsymbol{\alpha}$ is a vector of coefficients

- $\mathbf{z}(\mathbf{k}_c)$ is a vector of exposure-outcome, exposure-mediator, and mediator-outcome confounders measured without error, $k \in \{$2007 NDVI, LST, conflicts, disasters; 2009 conflicts, disasters$\}$
- P_c is the offset, given as population in county c
- S_c are county-level structured random effects which follow the ICAR model with marginal variance σ_s^2
- $ne(c)$ denotes neighbors (shared boundary) of county c
- n_c is the number of neighbors of county c
- ϵ_c are county-level iid (unstructured) random effects with variance σ_ϵ^2
- x_{lc} is the true livestock density in county c
- u_{lc} is the measurement error for livestock density in county c
- x_{wc} is the true wealth score in county c
- u_{wc} is the measurement error for wealth score in county c
- λ_l is the mean of the true livestock density
- λ_w is the mean of the true wealth score
- ζ_{lc} is the residual for livestock density in county c
- ζ_{wc} is the residual for wealth score in county c
- d_c is a weight which allows for heteroscedasticity in the error structure

For both livestock density and wealth score, we assume $x_c \sim N(\theta, \sigma_x^2)$, where θ is the mean of x_c and σ_x^2 is the variance.

There are therefore four parameters needed for each of the two resulting measurement error models: β_1 (β_2 for wealth score), $\log(1 / \sigma_u^2)$, λ, $\log(1 / \sigma_x^2)$, as well as the scale factor d_c. For livestock density, we specified the priors and starting values for these parameters as follows for livestock, substituting μ for η and β_1 for β_2 for wealth:

- $\log(1 / \sigma_u^2) \sim \text{logGamma}(10, 10)$, starting value $\log\big(1 / var(\sigma_\mu)\big)$
- $\beta_1 \sim \text{Normal}(\hat{\beta}_1, 1 / \hat{\sigma}^2_{\hat{\beta}_1})$.
- λ fixed at mean $E[\mu]$, with a Gaussian prior
- $\log(1 / \sigma_x^2) \sim \text{logGamma}(10, 10)$, starting value $\log(1 / var(\mu))$
- $d_c \sim \text{Unif}(0.5, 1.5)$

where $var(\sigma_\mu)$ is the empirical variance of the posterior standard deviation for livestock density, $\hat{\beta}_1$ is the posterior mean of the coefficient for livestock density from the naive model, $\hat{\sigma}_{\hat{\beta}_1}$ is the posterior standard deviation of the same coefficient from the naive model, and $var(\mu)$ is the empirical variance of the posterior median for livestock density. The logGamma prior specification is equivalent to that used in Wang et al. [1]. The MEC model for wealth was specified equivalently, and in our final models both livestock density and wealth were included in this form.

The MEC mediator model follows as for the outcome model detailed above.

BIBLIOGRAPHY

[1] Wang X, Yue YR, Faraway JJ. 10. In: Errors-in-Variables Regression. Boca Raton, FL: Chapman & Hall/CRC. Computer Science and Data Analysis Series; 2018. .
