# Supplementary material for: Livestock, pathogens, vectors, and their environment: A causal inference-based approach to estimating the pathway-specific effect of livestock on human African trypanosomiasis risk
Source: PLOS Glob Public Health. 2023 Nov 15;3(11):e0002543. doi: 10.1371/journal.pgph.0002543 (PMC10651035; doi:10.1371/journal.pgph.0002543)
Supplement: Attachment — Submitted filename: PLOS_GPH_RTR.docx [file pgph.0002543.s006.docx]

**Reviewer comments**

You use the term pigs here only in the context of domestic pigs and warthogs for wild pigs. Where are the bushpigs? Also later you just say pigs what do you actually mean. Domestic pigs are rare in South Sudan so not sure what is being examined there.

*We have added “bushpigs” to the list in the introduction. We have also clarified in the introduction that all analyses are only for domestic pigs, end of paragraph 1: “Hereafter, we use the term “livestock” to refer to cattle and domestic pigs.”*

*Regarding South Sudan, yes you are correct that domestic pigs are rare in many counties of South Sudan, though not all. This is captured in the livestock density estimates we created and cited (see Figure 16(b) for pigs in South Sudan):*

*Meisner, J., Kato, A., Lemerani, M. et al. A time-series approach to mapping livestock density using household survey data. Sci Rep* ***12****, 13310 (2022).* [*https://doi.org/10.1038/s41598-022-16118-1*](https://doi.org/10.1038/s41598-022-16118-1)

I am afraid presence or absence of reservoirs is poorly correlated with protected areas - for example buffalo distribution maps assumed on niche vegetation habitat data are highly unrealiable for a persecuted species and this amounts to virtual absence in some areas such as South Sudan even in so called protected areas. Whilst other species such as bushbuck are widely distributed and the PA model will be a poor representation of this species and of risk. Until assumptions are adjusted I feel there is a bias implicit in this modelling run. It may not make much difference but the authors should check.

*Thank you for raising this point, this is of course correct. We selected this metric as the best indicator (that we could think of) for wildlife density, but of course it’s an imperfect proxy. We added the following line to the limitations paragraph in the Discussion section:*

*Further, proximity to protected areas is an imperfect proxy for density of wildlife hosts, as wildlife can and do exist outside of protected areas.*

Might be worth defining at the first use what you mean by clusters and I am not sure this is done at any stage? For some readers this will be a confusion.

*We have added the following text to the start of the Materials & Methods section:*

*Cluster is defined as a 0.017° pixel.*
